# Breast Metastasis From Vulvar Carcinoma: Case Report and Review of Literature

**DOI:** 10.1155/crog/9936814

**Published:** 2025-12-26

**Authors:** Nayssem Khessairi, Saida Sakhri, Maha Chrigui, Ons Krimi, Hanen Bouaziz, Tarek Ben Dhiab

**Affiliations:** ^1^ Department of Surgical Oncology, Salah Azaiez Institute, Tunis, Tunisia; ^2^ Faculty of Medicine of Tunis, University of Tunis El Manar, Tunis, Tunisia, utm.rnu.tn

**Keywords:** breast cancer, diagnosis, metastatic, vulvar metastasis

## Abstract

**Introduction:**

Primary vulvar cancer is uncommon, accounting for only 3%–5% of all gynecological malignancies. Metastases to the vulva are even rarer, and those originating from breast cancer are exceptional, with fewer than 20 cases reported in the literature.

**Case Presentation:**

We report the first case observed at our institution. A 38‐year‐old woman had been treated for breast cancer. Ten years after completing treatment, she presented with an analgesic‐resistant headache, cervical swelling, and vulvar discomfort. Updated staging revealed hepatic, pulmonary, pleural, and bone metastases. Biopsy of the vulvar lesion confirmed metastasis of an infiltrating ductal breast carcinoma. The patient underwent chemotherapy, with disease progression despite treatment. She is currently receiving palliative chemotherapy.

**Conclusion:**

Early detection of unusual metastatic sites and appropriate management require careful monitoring of women with breast cancer. Pelvic and gynecological examinations should be included in the follow‐up of breast cancer patients to detect vulvar or vaginal metastases.

**Summary**

• Primary vulvar cancer is uncommon, accounting for only 3%–5% of all gynecological cancer.
• Tumors metastasizing to the vulva are even rare, and those originating from breast cancer are exceptional, with fewer than 20 cases reported in the literature.
• Treatment remains not codified given the rarity of the metastasis.

## 1. Introduction

Metastatic tumors involving the vulva are very uncommon, representing only 5%–8% of all vulvar tumors. The vulva is therefore a rare site of metastasis. Involvement of the vulva by breast cancer is an exceptional event [1, 2]. Breast cancer metastases usually spread via the lymphatic system, involving regional lymph nodes. Hematogenous spread typically affects the lungs, bones, liver, and brain [3]. Involvement of the female reproductive system is very rare. Although breast cancer may rarely involve the lower genital tract, the ovaries and uterus are the most frequently reported sites. Vulvar metastases from breast cancer are exceptional, with fewer than 20 cases reported in the literature, accounting for only 4% of cases [4].

Diagnosis and differentiation from primary vulvar cancer are challenging because these metastases are usually asymptomatic, and the diagnosis is often made incidentally during histological examination of gynecologic specimens resected for other reasons [4]. Optimal treatment remains unclear. Here, we report the first and unique case of vulvar metastasis from ductal carcinoma of the breast diagnosed in our country.

## 2. Case Presentation

A 38‐year‐old female patient without familial or medical history has been treated a decade earlier in our department for invasive ductal carcinoma of the left breast. The tumor measured 5 × 4 cm and an enlarged homolateral axillary lymph node, suspicious for metastasis, was present. No distant metastases were found. It was classified as T2N1M0. An immunohistochemical study showed estrogen and progesterone‐receptor positive, Ki67: 10% and the HER2neu was amplified. She underwent neoadjuvant chemotherapy in the form of four cycles of FEC (5‐fluorouracil, epirubicin, and cyclophosphamide) followed by four cycles of docetaxel, associated with trastuzumab‐based targeted therapy. Then, she underwent a conservative surgical treatment consisting of a tumorectomy with homolateral axillary lymph node dissection. The postoperative course was uneventful. Final histological examination revealed a 27‐mm invasive ductal carcinoma, SBR Grade II; the 13 lymph nodes were free of metastasis.

She also received adjuvant radiation therapy to the chest wall, followed by tamoxifen therapy. The patient discontinued tamoxifen therapy after 3 years and was lost to follow‐up. She re‐presented 5 years later, totaling 10 years after the initial breast cancer diagnosis, with analgesic‐resistant headache, cervical swelling, and vulvar discomfort due to a lesion on the labium majus.

Clinical examination revealed a suspicious 2‐cm supraclavicular adenopathy. No recurrence was observed in the left breast. Gynecological examination identified a small nodular lesion on the right labium majus. A full‐body scan revealed cerebral, hepatic, pulmonary, pleural, and bone metastases.

**Figure 1:**
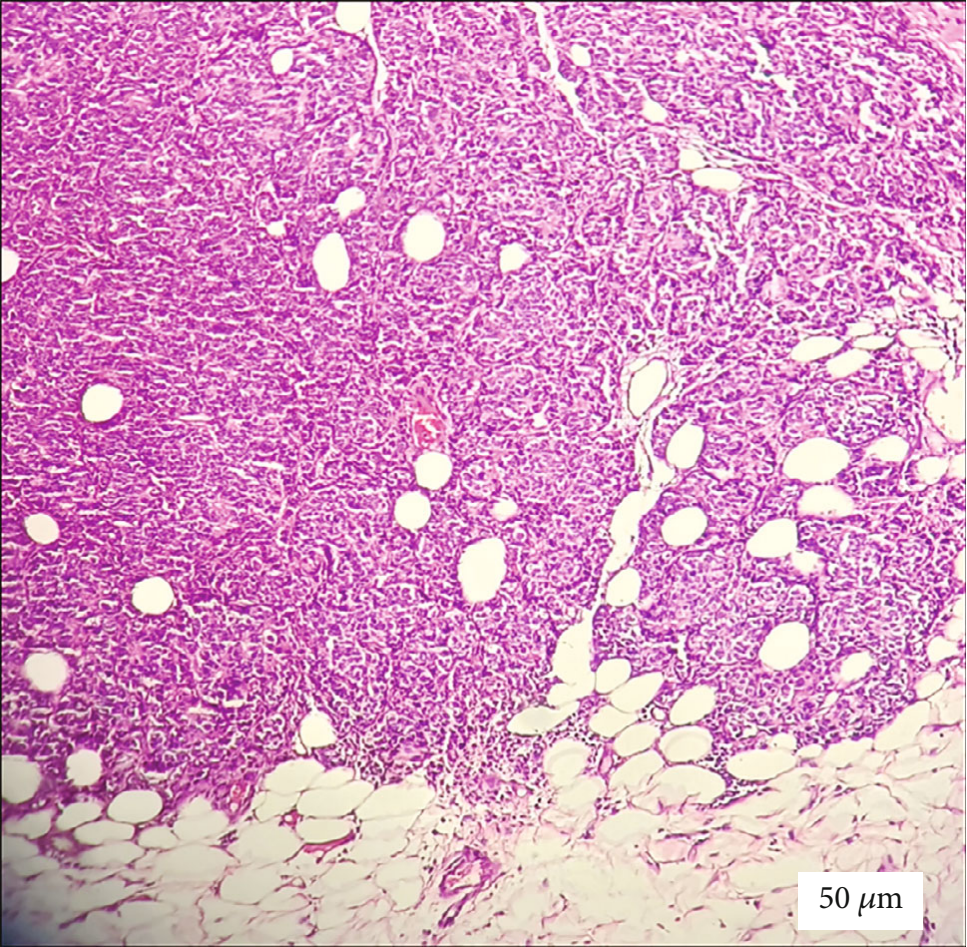
Histopathological examination of the vulvar lesion (H&E, 20× magnification) showing a solid proliferation of atypical epithelial cells, consistent with metastatic breast carcinoma. Scale bar = 50 *μ*m.

The vulvar lesion was biopsied to determine whether it represented a primary vulvar malignancy or a metastasis from her previous breast cancer. Histopathological examination showed a solid proliferation of atypical epithelial cells, consistent with metastatic breast carcinoma. The tumor cells were arranged in solid sheets rather than cords of single cells. Immunohistochemical analysis demonstrated positive estrogen receptor expression, negative progesterone receptor expression, and absence of HER2 protein overexpression (score 1+) (Figure 1).

The multidisciplinary committee recommended palliative chemotherapy with capecitabine (Xeloda). The patient experienced disease progression under this regimen, and a second line of chemotherapy with cyclophosphamide, methotrexate, and fluorouracil (CMF) was initiated. The patient is currently receiving palliative chemotherapy.

## 3. Discussion

The vulva is a rare site of metastasis within the female genital tract [1]. To date, data on this topic are limited, and the available literature mainly consists of isolated case reports. Metastases from breast cancer to the vulva are even rarer. Although breast cancer is the most common malignancy in women, vulvar metastases are exceptional. The most frequent sites of breast cancer metastases are the lungs, liver, bones, and brain, whereas vulvar involvement is extremely uncommon [3].

The exact mechanism underlying this metastatic pattern is not clearly understood, although involvement of vascular spaces has been suggested as a possible cause. Altered lymphatic drainage after surgery may also contribute to this pattern. Valenzano et al. reported a case in which a 49‐year‐old patient developed a rectus abdominis myocutaneous flap metastasis 3 years after surgery, followed by a vulvar metastasis 11 years after the primary surgery. The authors hypothesized that lymphatic spread through newly formed lymphatic channels might have occurred [2].

In the studies by Lamovec and Borst, invasive lobular carcinoma was identified as the most frequent histological type involving the female genital tract, including the ovaries, uterus, and vagina; however, vulvar involvement was not reported in either study [5, 6].

We conducted a literature review, which identified fewer than 20 reported cases of vulvar metastases from breast cancer, summarized in Table 1. Vulvar metastases from breast cancer are usually asymptomatic. The clinical presentation is variable and can mimic benign lesions such as vulvar dermatitis or abscesses, making diagnosis challenging for clinicians [13].

**Table 1:** Reported cases of breast cancer metastatic to the vulva.

**Authors/year**	**Age (y/o)**	**Histological type**	**Delay to metastasis (months)**	**Other metastases**
1. Covington 1964 [7]	78	Comedocarcinoma	66	N/A
2. Patsner 1996 [8]	N/A	N/A	N/A	N/A
3. Curtin and Murthy 1997 [9]	61	IDC	4	N/A
4. Menzin et al. 1998 [10]	53	ILC	Synchronous	N/A
5. Sindico et al. 1998 [11]	79	IDC	144	N/A
6. Porzio et al. 2001 [12]	67	IDC	168	N/A
7. Miliaras 2002 [13]	45	IDC	12	N/A
8. Neto et al. 2003 [2]	65	IDC	24	Yes
	48	ILC	84	Yes
	47	Undifferentiated	60	Yes
	53	Cystosarcoma phyllodes	48	Yes
9. Valenzano et al. 2003 [14]	49	N/A	132	N/A
10. Sheen‐Chen et al. 2004 [15]	32	ILC	40	N/A
11. Perrone et al. 2009 [16]	72	IDC	Synchronous	N/A
12. Papaioannou et al. 2010 [17]	93	ILC	156	No
13. Julien et al. 2012 [18]	68	IDC	72	Yes
14. Alligood‐Percoco et al. 2015 [19]	57	ILC	255	Yes
15. Gandhi et al. 2015 [20]	76	IDC	168	Yes
16. Doval et al. 2020 [21]	50	IDC	48	Yes
17. Our case 2024	28	IDC	120	Yes

Abbreviations: IDC, infiltrative ductal carcinoma; ILC, infiltrative lobular carcinoma; N/A, not applicable.

Accurate diagnosis requires a multidisciplinary approach, including clinical examination, radiological investigations, histopathology, and immunohistochemistry. The possibility of vulvar metastasis should be considered, particularly in patients with a prior history of breast cancer. Identical histological features and hormone receptor status between the primary breast tumor and the vulvar lesion can aid in confirming the diagnosis.

According to the literature, the most common clinical presentation is the perception of a mass or swelling in the vulvar region [15, 18, 19]. Other reported symptoms may include pain, itching, bleeding, or ulceration of the vulva [20, 21]. Our patient presented after 10 years after the treatment of breast cancer with a suspicious vulvar lesion without revealing symptoms. The occurrence of a vulvar metastasis in breast cancer typically indicates advanced disease with diffuse multivisceral metastases [2, 4].

There is no consensus on the management of this type of metastasis. Previous studies have suggested that treatment should follow the same principles as for other visceral metastases of breast cancer. Surgical resection of the tumor is recommended when feasible, particularly in cases of infected or bleeding lesions to achieve local control of the tumor, and should ideally be followed by systemic therapy. Treatment often involves chemotherapy and/or hormone therapy; however, it is frequently palliative and primarily based on chemotherapy [18]. Decisions regarding management should be made by a multidisciplinary team and typically depend on the patient’s general condition as well as the extent and location of metastases. In cases of controlled disease or a single metastatic site, radical vulvectomy may be considered. The extent of the surgical procedure depends on the size and location of the metastasis [20].

In our patient, multiple metastatic sites beyond the vulva were present. Therefore, surgical excision of the vulvar lesion was not proposed, and systemic treatment with chemotherapy was initiated. Radiation therapy may be used as an adjuvant treatment after surgery or as a primary treatment for patients with unresectable vulvar metastases [2]. The prognosis for patients with vulvar metastasis from breast cancer depends on the presence of other metastases, their extent, and the response to treatment. In general, it indicates diffuse, advanced disease with a poor prognosis and a 5‐year survival rate less than 20% [12, 17]. Finally, we should keep in mind that there is another diagnosis to consider, despite its rarity: the primary mammary‐like adenocarcinomas of the vulva (MLAV) which share histological, immunohistochemical, and molecular similarities with breast carcinoma. Treatment strategies are largely extrapolated from breast cancer management. The prognosis is better than this case of vulvar metastasis from breast cancer [22].

## 4. Conclusion

Early detection of unusual metastatic sites and their appropriate management require careful monitoring of women with breast cancer. Pelvic and gynecological examinations are recommended as part of the follow‐up for breast cancer patients. This is particularly important not only for patients receiving adjuvant hormone therapy (e.g., tamoxifen), where iatrogenic endometrial carcinoma is not uncommon, but also for other patients, in accordance with ASCO (American Society of Clinical Oncology) guidelines. The optimal duration of surveillance in breast cancer patients in remission remains controversial. This case report, along with others cited in the literature, supports long‐term, and even indefinite, follow‐up with the goal of detecting metastatic disease as early as possible.

## Ethics Statement

The authors declare that this work was done with all due respect to the code of ethics under the supervision of the medical and ethics committee of the Salah Azaiez Institute.

## Consent

Written informed consent was not required as no identifiable information is included.

## Disclosure

All authors read and approved the final manuscript.

## Conflicts of Interest

The authors declare no conflicts of interest.

## Author Contributions

N.K. wrote the main manuscript. S.S. collected the data and drafted the manuscript. M.C. collected the data and reviewed the literature. T.B.D. drafted the manuscript. O.K. prepared figures.

## Funding

No funding was received for this manuscript.

## Data Availability

The data that support the findings of this study are available from the corresponding author upon reasonable request.
